# Comparing safety and efficiency of two closed-chamber techniques for iridodialysis repair - a retrospective clinical study

**DOI:** 10.1186/s12886-018-0984-y

**Published:** 2018-12-07

**Authors:** Wenjuan Wan, Lei Shi, Can Li

**Affiliations:** 1 Chongqing Key Laboratory of Ophthalmology and Chongqing Eye Institute, The First Affiliated Hospital of Chongqing Medical University, Chongqing, 400016 People’s Republic of China; 2 The Fifth Affiliated Hospital of Zhengzhou University, Zhengzhou, 450052 People’s Republic of China

**Keywords:** Iridodialysis, Iridoplasty, Closed-chamber technique, Iridodialysis repair, Iris tear

## Abstract

**Background:**

This study aims to compare the safety and effectiveness of two closed-chamber techniques for repairing iridodialysis.

**Methods:**

Seventy five patients with iridodialysis undergoing surgery from February 2008 to October 2017 were included in this study. Patients were divided into two Groups, Group A (32 eyes) and Group B (35 eyes), with Group A using a 26-gauge hypodermic needle guided 10–0 nylon suture, and Group B using a double-armed polypropylene suture. Before operation and 1, 3, and 6 months after the operation, pupil shape, best corrected visual acuity (BCVA), intraocular pressure (IOP), the rate of endothelial cell loss, and intra- and postoperative complications were compared between two Groups during the follow-up period.

**Results:**

Iridodialysis was repaired with pupil shape restored in all cases. IOP was normalized in all eyes except 2 eyes (6.3%) in Group A and 3 eyes (8.6%) in Group B. Postoperative rate of endothelial cell loss was not significantly different between two Groups (*P* > 0.05). The percentage of complicated cataract was not significantly different in Group A (2 eyes, 6.3%) compared to Group B (2 eyes, 5.7%) (χ2 = 0.009, *P* = 0.658).

**Conclusions:**

Both techniques for repairing iridodialysis not only were safe but also effective in improving visual function and cosmetic recovery. However, double-armed polypropylene suture might be less invasive than 26-gauge hypodermic needle guided suture.

## Background

Iridodialysis is a separation of the iris from its attachment to the ciliary body. It commonly occurs secondary to blunt or penetrating ocular trauma, and intraocular surgical procedures, in which ocular contusion is the most common cause. [1, 2] It was reported that the percentage of iridodialysis in blunt injury was 9.3% [3], while that occurred in 0.2% of patients who underwent cataract surgeries. [4] Surgical repair should be carried out only if iridodialysis is associated with symptoms, such as monocular diplopia, photophobia, glare, accompanying with problems that unable to perform additional maneuvers in the iris or cosmetic deformations (e.g., polycoria and ectopic pupil).

Various surgical techniques have been used to restore the anatomy of iris, including closed [5–7] and open chamber [8] iridoplasty. Since the restored iris root was attached inside of the sclera incision in open chamber iridoplasty, reconstruction of closed chamber iris was proved to be safer and less invasive than open chamber techniques. [9] 26-gauge needle guided 10–0 nylon suture and double-armed polypropylene suture were two common techniques for closed chamber iridoplasty. [10–12] However, which technique offers better clinical remains unknown. Therefore, we here attempted to compare these two surgical techniques to determine their safety and effectiveness in treating iridodialysis.

## Methods

A retrospective cohort study was performed on the Department of Ophthalmology, First Affiliated Hospital of Chongqing Medical University (Chongqing, China). 75 patients with iridodialysis who underwent surgery from February 2008 to October 2017 were included in this study. Inclusion criteria were as follows: (1) The range of iridodialysis was more than 90 degrees; (2) Patients complained about monocular diplopia, photophobia, or glare; (3) Patients were unable to perform additional maneuvers in the iris; (4) Cosmetic problems (e.g., polycoria and ectopic pupil) were present. The study was approved by the Institutional Review Board, First Affiliated Hospital, Chongqing Medical University.

Age, gender, diagnosis, and treatment were recorded in the hospital’s database. The following were thoroughly asseses in each patient before surgery and after surgery (1, 3 and 6 months): best corrected visual acuity (BCVA, that was monocularly assessed and recorded as LogMAR scores by using the standard logarithmic visual acuity chart), intraocular pressure (IOP), slit-lamp examination of the anterior and posterior segment, and complications. Postoperative gonioscopy was used to identify whether the iris root was reattached or anterior peripheral synechiae occurred. Minimal clinically important improvement (MCII) was defined as an improvement of more than 2 lines on the visual acuity chart. Corneal endothelium cell was counted to analyze the rate of endothelial cell loss.

**Fig. 1:**
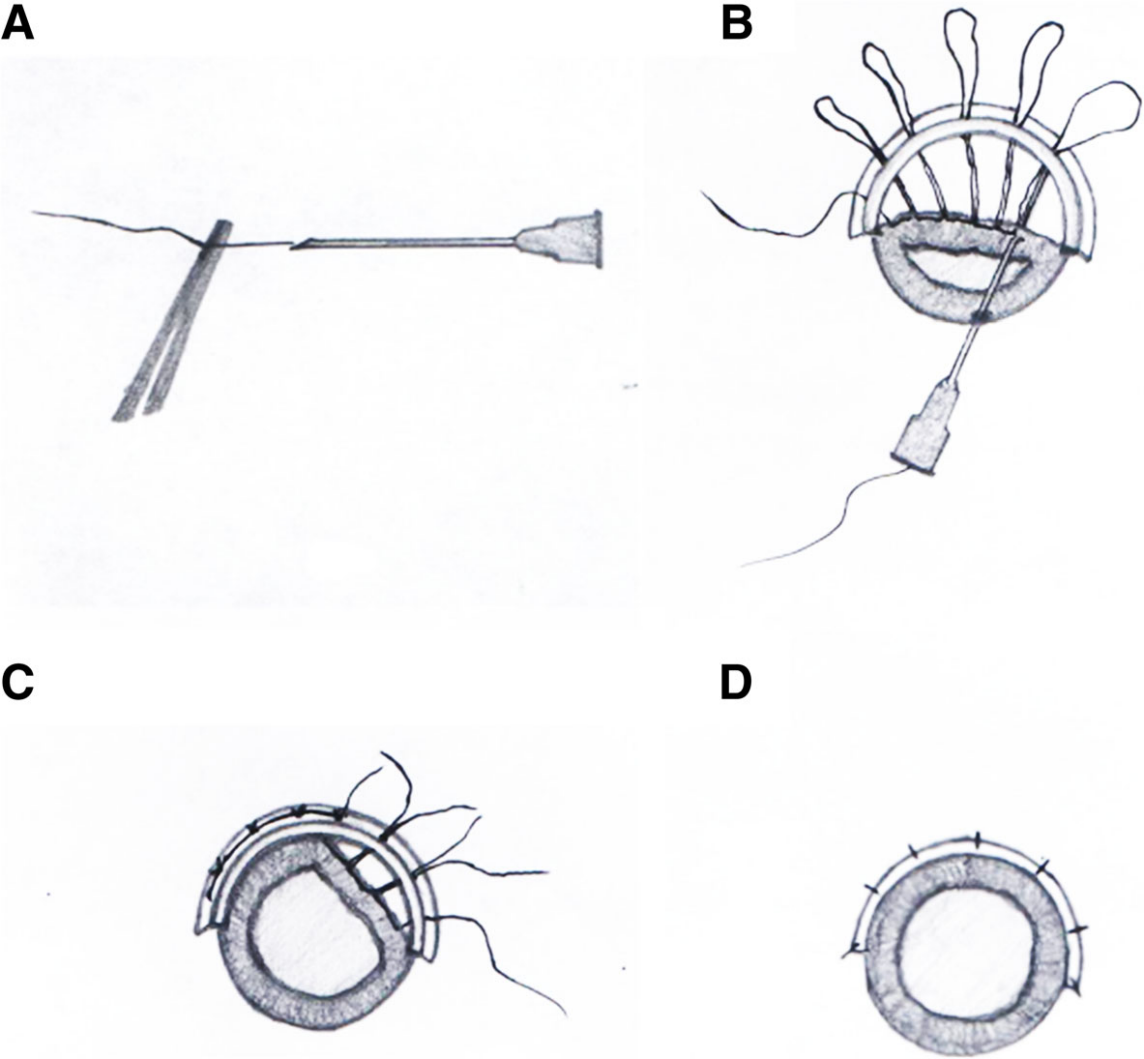
Procedure using a 26-gauge hypodermic needle guided 10–0 nylon suture for iridodialysis repair. **a**: a 26-gauge needle was threaded with a 10–0 nylon suture. **b**: the needle with suture was inserted in the anterior chamber through the limbus, then pushed forward to engage the disinserted iris root, then brought it out through the scleral bed, the free end of the suture was pulled out, and the needle was retracted into the anterior chamber. The procedure was repeated at another point, and the suture was pulled out to form a loop. **c**: the loop of a suture was cut, and the ends were tied to pull the iris back. **d**: the partial-thickness scleral flap was sutured

**Fig. 2:**
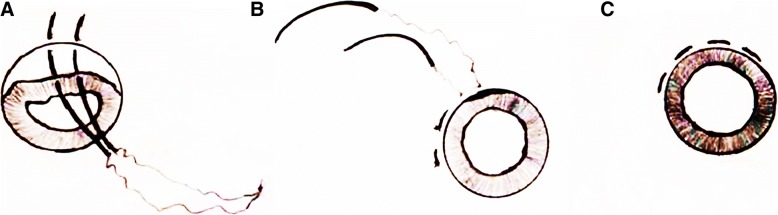
Procedure using a double-armed polypropylene suture for iridodialysis repair. **a**: one end of a double-armed 10–0 polypropylene suture on a curved needle was introduced into the anterior chamber via a paracentesis. The needle was driven through the iris base and penetrated out through the sclera (1 mm), which was posterior to the limbus. Then, the second arm was introduced into the anterior chamber via the same paracentesis and passed through the second point of the iris base. The suture was tied at the paracentesis side. **b**: the knot was pulled out through one of the sclera tunnels without scleral flap. The iris was pulled back, while polypropylene suture was fixed on the scleral by the iridodialysis side. **c**: the knots were rotated within the scleral needle tract, and the procedure was repeated until the iris was restored

These two closed-chamber iridodialysis repair procedures are both standard procedures in our hospital. Patients provided informed consent and chose the techniques. Patients were divided into two Groups according to different surgical techniques. In Group A, a 10–0 polypropylene suture was threaded through a 26-gauge needle, while in Group B, patients underwent double-armed polypropylene suture. [11] In Group A, a partial-thickness scleral tunnel was created 1.5 mm from the limbus along the extent of the iridodialysis. A 26-gauge needle threaded with 10–0 nylon suture was inserted into the anterior chamber through the limbus (180 degrees far from the iridodialysis), then pushed forward to engage the disinserted iris root and brought it out of the eye within the scleral bed. The free end of the suture was pulled out, and the needle was withdrawn into the anterior chamber with the suture remaining in the lumen of the needle. The procedure was repeated at another point, and the suture was pulled out to form a loop. These steps were repeated multiple times depending on the size of the iridodialysis until multiple loops laid over the scleral, approximately 2 sutures every quarter. The suture was cut, the ends were tied, the partial-thickness scleral flap was sutured, and the conjunctival peritomy was closed (Fig. 1). In Group B, a paracentesis was created on the opposite side of the cornea. One end of a double-armed 10–0 polypropylene suture on a curved needle was introduced into the anterior chamber via the paracentesis. The needle was driven through the iris base and penetrated out through the sclera 1 mm posterior to the limbus. After that, the second arm was introduced into the anterior chamber by the same paracentesis and passed through the second point of the iris base. The suture was tied at the paracentesis side, and the knot was pulled out through one of the sclera tunnels without scleral flap, to ensure scleral fixation. The procedure was repeated until the iris was restored [12] (Fig. 2). All the surgeries were performed under the same viscoelastic material (Medical sodium hyaluronate gel for ophthalmology, purchased from Shenyang Sinqi Pharmaceutical Co., Ltd., China) to maintain the stability of the anterior chamber.

### Statistical analysis

Data analysis was performed using SPSS 16.0 software (SPSS Inc., Chicago, IL, USA). Results were expressed as the mean ± standard deviation (SD). The independent-samples *t*-test was used to compare the variables. The paired sample *t*-test was used to compare the paired variables. Categorical variables were analyzed using two-tailed Pearson’s chi-squared test. *P*-value < 0.05 was considered statistically significant.

### Results

### General information

A total of 75 iridodialysis patients (75 eyes) were admitted to our ophthalmology department during the study period. 35 and 40 patients were assigned to Group A and Group B, respectively. However, 3 cases in Group A and 5 cases in Group B were excluded because the follow-up period was less than 6 months. Eventually, 32 and 35 patients were included in Group A and Group B, respectively (Table 1). In Group A, there were 30 males and 2 females, with ages of 32.3 ± 11.0 years. In Group B, there were 33 males and 2 females, with ages of 30.3 ± 11.2 years. Neither the gender nor age was different between these two groups. All of the investigated patients were followed up for 6 to 96 months, with a mean follow-up duration of 27.8 ± 12.3 months.

**Table 1:** Demographic data of study subjects

	Group A	Group B	*P*-value	Total
Patients (N)	32	35	N/A	67
Eyes studied (n)	32	35	N/A	67
Gender (Male/Female)	30/2	33/2	0.658	63/4
Age (years)	32.3 ± 11.0	30.3 ± 11.2	0.479	
Causes				
Contusion (n)	31	34	0.731	65
Open globe(n)	1	1		2
Degree of iridodialysis				
< 1 quadrant(n)	11	9	0.821	20
1–2 quadrant(n)	12	15		27
> 2 quadrant(n)	9	11		20
Surgical procedure				
Combined with lens removal (n)	20	17	0.455	37
Combined with anterior vitrectomy and lens removal(n)	6	7	0.572	13

Contusion was found to be the major cause of iridodialysis. A total of 37 eyes (20 eyes in Group A and 17 eyes in Group B) were accompanied with complicated cataract and/or lens subluxation that led to a remarkable visual impairment, therefore lens removal was performed with iridodialysis repair. 13 eyes (6 eyes in Group A and 7 eyes in Group B) underwent anterior vitrectomy because vitreous prolapsed into the anterior chamber with lens dislocation. The demographic data such as gender, age, causes of the disease, the degree of iridodialysis and number of cases accompanied with lens dislocation and anterior vitrectomy were not significantly different between Group A and Group B (Table 1).

### Efficiency of the two techniques for repairing iridodialysis repair

**Fig. 3:**
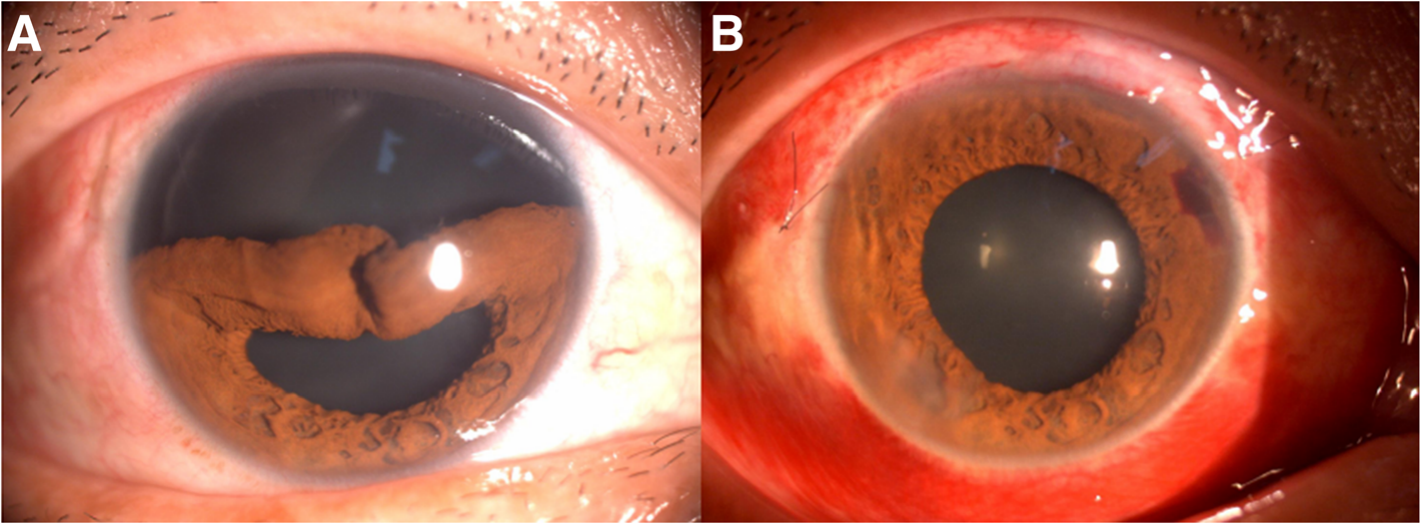
Preoperative and postoperative slit-lamp photographs of iridodialysis patients who underwent 26-gauge hypodermic needle guided 10–0 nylon suture. **a**: preoperative appearance with iris dislocated between 9 o’clock to 3 o’clock, and the pupil shape became crescent. **b**: illustration of pupil restored to nearly round shape at 1 day after operation

**Fig. 4:**
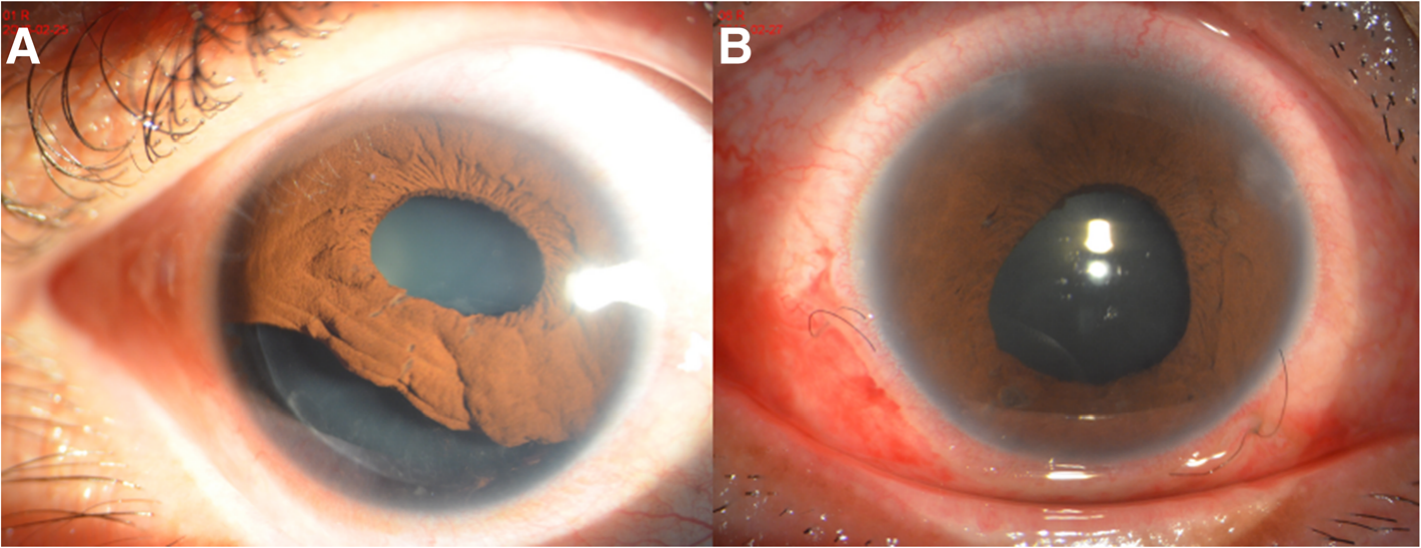
Preoperative and postoperative slit-lamp photographs of iridodialysis patients who underwent double-armed polypropylene suture. **a**: preoperative appearance with iris dislocated between 4 to 9 o’clock. **b**: illustration of pupil restored to nearly round shape at 1 day after operation

The pupil shape restored to round or nearly round in all cases at 1, 3, and 6 months after the operation. Gonioscopy showed that the iris root was reattached. The pupil shape was adjusted by a slit-lamp examination. In Group A, pupil shape recovered to round shape in 15 eyes (46.9%), and nearly round shape in 17 eyes (53.1%), as shown in Fig. 3. While in Group B, pupil shape recovered to round shape in 14 eyes (40.0%), and nearly round shape in 21 eyes (60.0%), as illustrated in Fig. 4. There were no significant differences between two Groups in terms of the reconstruction of pupil shape (χ^2^ = 0.322, *P* = 0.627).

A higher percentage of iris tear was found in Group A (9 eyes, 24.1%) compared to Group B (0 eyes) (χ^2^ = 11.371, *P* = 0.001). Minor iris tear around the access passage to needle was detected in Group A, especially when the range of iridodialysis was wide (in 6 cases the degree of iridodialysis was higher than 2 quadrants), and/or with mild iris atrophy (2 cases). Hyphema caused by iris trauma was not observed as well. However, iris tear was not observed in Group B.

Limitation of vision acuity was not closely associated with iridodialysis. Improvement of visual function was found in 24 eyes (78.1%) in Group A and 26 eyes (74.3%) in Group B at 1 month after the operation, without significant differences between the two Groups (χ2 = 0.005, *P* = 0.585). After 6 months of follow-up, visual function was eventually improved in all patients except for 2 eyes (optic atrophy and vitreous opacity) in Group A and 1 eye (a choroidal rupture) in Group B. Improvement of visual function not only was achieved by restoring the iris anatomy. Cataract and lens dislocation were also effectively treated.

### Safety of the two techniques for repairing iridodialysis

IOP became normal at 1, 3, and 6 months after the operation, except 2 eyes (6.3%) eyes in Group A and 3 eyes (8.6%) eyes in Group B complicated with angle recession. There was no significant difference between these two Groups (χ2 = 0.130, *P* = 0.543). The rate of endothelial cell loss before and after the operation was not significantly different between Group A and Group B (Table 2).

**Table 2:** Comparing mean corneal endothelium cell count and the corresponding loss rate between group A and group B before and after the operation

Mean Corneal Endothelium Cell Count and loss rate (/mm^2^,%)	Pre-operation	1 month after operation	3 months after operation	6 months after operation
Group A	2479.3 ± 226.5	2122.6 ± 186.7,14.4	2273.8 ± 211.4,8.3	2347.4 ± 219.2,5.3
Group B	2386.8 ± 235.2	2117.4 ± 205.1,11.3	2196.2 ± 202.8,8.0	2232.9 ± 207.7,6.5
*P-value*		*0.262*	*0.577*	*0.582*

The percentage of complicated cataract was not significantly different between Group A (2 eyes, 6.3%) and Group B (2 eyes, 5.7%) (χ2 = 0.009, *P* = 0.658). Besides, cataract surgery was not performed, as the lens opacity was not severe enough to greatly affect visual acuity. Hyphema was observed in 5 eyes (15.6%) in Group A, and 1 eye (5.9%) in Group B without observing significant differences between these two Groups (χ2 = 3.342, *P* = 0.096). Hyphema was absorbed 7 days after surgery without complications. No other complications were found during the follow-up period as well.

## Discussion

The iris root is one of the weakest parts of iris that could be easily impaired. [12, 13] In the present study, contusion is the most common cause of iridodialysis. Two closed chamber techniques for iridodialysis repair have been shown to be safe and effective, although minor iris tear may be caused by a 10–0 polypropylene suture that was threaded through a 26-gauge needle.

Different techniques including open-chamber [8, 9] and closed-chamber [11, 12] approaches for surgical repair of traumatic iridodialysis have been reported. After Paton presented an incarceration technique in 1973 [8], McCannel [9] introduced a surgical method that uses a curved needle and a 10–0 nylon suture. Both methods were open-chamber techniques. As the iris was squeezed between the sclera lips, open-chamber technique is not precise in restoring the anatomy of iris, and may increase the risk of infection, [6] and in some rare cases, cause epithelialization of the anterior chamber . [13] A number of closed-chamber technique has been reported as well. For instance, Nunziata [11] used a 17.0 mm straight needle and a 10–0 polypropylene suture. However, the 17.0 mm straight needle did not have a handle, thus manipulation is difficult and the needle may easily damage the crystalline lens. A curved needle would potentially avoid the above mentioned problems. Bardak [6] used a 22.0 mm 26-gauge straight needle with a hole (1.0 mm) from the tip to insert a 10–0 suture which was similar to ours, however, scleral flap might decrease the risk of delayed-onset intraocular infection including endophthalmitis. [14] In addition to these techniques, ‘hang back’ surgical approach was proposed to titrate the tightness of anchoring of the suture [15] in order to facilitate manipulation by using a 27 gauge needle [12]. Knotless technique [16] and single-tread single-knot suture [5] are two other techniques. Two approaches were chosen in our study: 1) 26-gauge straight needle guided suture with scleral flap, and 2) double-armed 10–0 polypropylene suture. Both approaches were proved safe and effective methods during a 6-month follow-up period.

Minor iris tear around the area where the needle passes through was observed in certain patients who underwent 26-gauge needle guided suture, but not in those treated with a double-armed polypropylene suture with a curved needle. The outer diameter of a 26-gauge hypodermic needle was 0.45 mm, while the cross-section of the double-armed curved needle was 0.2 × 0.1 mm^2^. The puncture area on the iris made by a 26-gauge needle was larger than that created by a double-armed curved needle. Thus, weaker or atrophic peripheral iris might be more likely to be avulsed by a 26-gauge needle. Bhende [7] used a 30-gauge hypodermic needle (outer diameter = 0.3 mm), which might be less invasive than a 26-gauge needle, however, the cross-section area was still larger than that of double-armed curved needle. Complications such as hyphema was not observed, and the pupil shape and visual function were not affected either.

There are several limitations in our study: it is a retrospective study, and the sample size was small as well. The minimum or maximum mesopic size of pupil was not recorded by objective anterior eye segment analysis system, such as corneal topography or anterior eye segment tomography. Subjective discomfort including glare pre- and post- operation that might be relevant to the extent of dialysis was not analyzed in the study. A longer duration of follow-up, objective analyses of post-operative pupil size, as well as subjective evaluation should be considered in the future prospective studies.

## Conclusions

Our study compared two closed-chamber approaches for iridodialysis repair. The results revealed that both methods were safe and effective in correcting the functional and cosmetic problems.

## Abbreviations

BCVA: Best corrected visual acuity
BCVA: Intraocular pressure
MCII: Minimal clinically important improvement
